# Carotenoid Biosynthesis in Oriental Melon (*Cucumis melo* L. var. *makuwa*)

**DOI:** 10.3390/foods8020077

**Published:** 2019-02-19

**Authors:** Pham Anh Tuan, Jeongyeo Lee, Chang Ha Park, Jae Kwang Kim, Young-Hee Noh, Yeon Bok Kim, HyeRan Kim, Sang Un Park

**Affiliations:** 1 Department of Crop Science, Chungnam National University, 99 Daehak-ro, Yuseong-gu, Daejeon 34134, Korea; tuan_pham_6885@yahoo.com (P.A.T.); parkch804@gmail.com (C.H.P.); 2 Plant Systems Engineering Research Center, Korea Research Institute of Bioscience and Biotechnology (KRIBB), 125 Gwahangno, Yuseong-gu, Daejeon 305-806, Korea; leejy@kribb.re.kr (J.L.); yhnoh87@kribb.re.kr (Y.-H.N.); 3 Division of Life Sciences, College of Life Sciences and Bioengineering, Incheon National University, Yeonsu-gu, Incheon 406-772, Korea; kjkpj@inu.ac.kr; 4 Department of Medicinal and Industrial Crops, Korea National College of Agriculture & Fisheries, 1515, Kongjwipatjwi-Ro, Jeonju, Jeonbuk 54874, Korea; yeondarabok@korea.kr; 5 Systems and Bioengineering, University of Science and Technology, 217 Gajung-ro, Daejeon 34113, Korea

**Keywords:** β-carotene, carotenoids, *Cucumis melo* L. var. *makuwa*, chamoe, gene characterization, lutein

## Abstract

Full-length cDNAs encoding ξ-carotene desaturase (CmZDS), lycopene ε-cyclase (CmLCYE), β-ring carotene hydroxylase (CmCHXB), and zeaxanthin epoxidase (CmZEP), and partial-length cDNA encoding ε-ring carotene hydroxylase (CmCHXE) were isolated in Chamoe (*Cucumis melo* L. var. *makuwa*), an important commercial fruit. Sequence analyses revealed that these proteins share high identity and common features with other orthologous genes. Expression levels of entire genes involved in the carotenoid biosynthetic pathway were investigated in the peel, pulp, and stalk of chamoe cultivars Ohbokggul and Gotgam. Most of the carotenoid biosynthetic genes were expressed at their highest levels in the stalk, whereas carotenoids were highly distributed in the peel. The expression levels of all carotenoid biosynthetic genes in fruits of the native cultivar Gotgam chamoe were higher than those in the cultivar Ohbokggul chamoe, consistent with the abundant carotenoid accumulation in Gotgam chamoe fruits and trace carotenoid content of Ohbokggul chamoe fruit. Lutein and β-carotene were the dominant carotenoids; high levels (278.05 μg g^−1^ and 112.02 μg g^−1^ dry weight, respectively) were found in the peel of Gotgam chamoe. Our findings may provide a foundation for elucidating the carotenoid biosynthetic mechanism in *C*. *melo* and inform strategies for developing new chamoe cultivars with improved characteristics.

## 1. Introduction

Melon (*Cucumis melo* L.), which belongs to the Cucurbitaceae family, is one of the most highly consumed fruit crops worldwide because of its pleasant flavor and nutritional value. Melons provide a rich source of protein, minerals, vitamins, and a wide range of antioxidant compounds [1,2,3]. The fruit can be consumed as a salad or as juice and is used by the food industry in products such as jam, ice cream, and yogurt. Melon fruits are diverse in shape, size, color, and flavor. In Korea, oriental melon (*Cucumis melo* L. var. *makuwa*), commonly known as chamoe, is an important commercial fruit due to its vigorous growth, good quality, and unique flavor, and consumer demand for the fruit is high. Chamoe has also been used in traditional medicine as a liver tonic and for its cardio-protective, antidiabetic, anti-obesity, and anticancer properties [4,5]. The cultivar Ohbokggul chamoe, which has a golden-colored skin with silver lines and sweet white flesh, is one of the most popular fruits on the market. The native Korean cultivar chamoe, Gotgam, has green skin with distinctive green stripes running from end to end, and very thick, light-green flesh. Gotgam chamoe has greater flavor, nutrient content, and disease resistance than other chamoe cultivars [6]. Given these favorable characteristics, Gotgam chamoe has recently acquired agronomic relevance for melon breeding programs in Korea.

Carotenoids, which contain 40 carbon molecules and are formed through the condensation of isoprenoids, represent a diverse group of pigments in nature [7]. In plants, carotenoids contribute to yellow, orange, and red coloration, and play a major role in the quality of flowers and fruit. The biosynthesis of biomolecules as carotenoids could be related to agri-environmental factors [8]. Several studies have demonstrated a positive correlation between phytochemical biosynthesis and light intensity and the spectral quality of vegetables and microgreens produced in controlled environments [9]. Carotenoids are accessory pigments that harvest light for photosynthesis, protect the photosystem from photooxidation, and attract pollinators and agents of seed dispersal [10,11,12]. In addition, the oxidative cleavage of carotenoids produces apocarotenoids, which serve as development signals and antifungal agents and contribute to the flavor and aroma of flowers and fruit [13]. In terms of human health, carotenoids play an important protective role as antioxidants, and a diet containing carotenoid-rich vegetables and fruit can reduce the risk of cancer, cardiovascular disease, macular degeneration, cataracts, and ultraviolet-induced skin damage [14,15,16,17]. More than 50 carotenoids with β-ring end groups (e.g., β-carotene and β-cryptoxanthin) are precursors of vitamin A, which is one of the most important micronutrients affecting human health [18,19]. Vitamin A deficiency increases the risk of infectious disease, especially measles, diarrhea, and malaria, and is considered the most common public health problem among preschool-aged children [20,21]. The importance of carotenoids to human health has led to an increase in studies of vegetables and fruit that contain these compounds.

**Figure 1:**
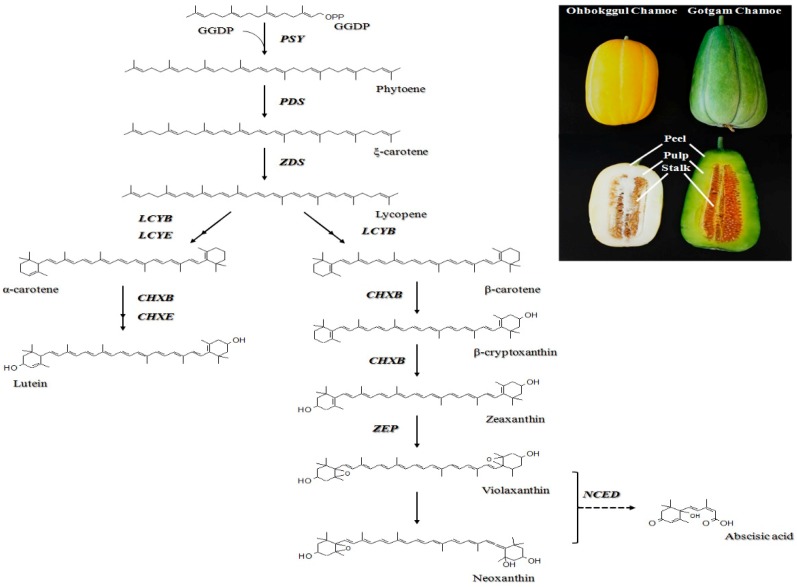
Carotenoid biosynthetic pathway in plants and photographs of Ohbokggul and Gotgam chamoes. GGDP (geranylgeranyl diphosphate); PSY (phytoene synthase); PDS (phytoene desaturase); ZDS (ξ-carotene desaturase); LCYB (lycopene β-cyclase); LCYE (lycopene ε-cyclase); CHXB (β-ring carotene hydroxylase); CHXE (ε-ring carotene hydroxylase); ZEP (zeaxanthin epoxidase); NCED (9-*cis* epoxycarotenoid dioxygenase).

In higher plants, carotenoids are synthesized and localized in the plastids, while the corresponding genes are located in the nucleus. To date, genes involved in carotenoid biosynthetic pathways in higher plants have been described in detail [22]. The first step in the formation of carotenoids is the condensation of two geranylgeranyl diphosphate (GGDP) molecules to form phytoene, which is catalyzed by phytoene synthase (PSY) (Figure 1). Phytoene undergoes a series of four desaturations to form lycopene via ξ-carotene, which is catalyzed by two enzymes, phytoene desaturase (PDS) and ξ-carotene desaturase (ZDS). Lycopene is a branching point in the pathway and is cyclized to form α-carotene by lycopene β-cyclase (LCYB) together with lycopene ε-cyclase (LCYE) or to produce β-carotene by LCYB alone through two reactions. Thereafter, α-carotene and β-carotene are hydroxylated to produce lutein and zeaxanthin, respectively. These reactions are catalyzed by β-ring carotene hydroxylase (CHXB) and ε-ring carotene hydroxylase (CHXE). Further epoxidation of zeaxanthin by zeaxanthin epoxidase (ZEP) produces violaxanthin, which is used to synthesize plant hormone abscisic acid (ABA) through oxidative cleavage catalyzed by 9-*cis* epoxycarotenoid dioxygenase (NCED) [23]. Along the pathway, carotenoids can be cleaved by carotenoid cleavage dioxygenases (CCD) to produce a diverse set of apocarotenoids [13].

Here, full-length cDNAs encoding ZDS, LCYE, CHXB, and ZEP, and partial-length cDNA encoding CHXE were isolated in *C. melo*. The expression levels of genes involved in carotenoid biosynthesis and carotenoid accumulation were investigated in fruits of the cultivar Ohbokggul and the native cultivar Gotgam chamoe using quantitative real-time PCR and high-performance liquid chromatography (HPLC), respectively. Therefore, this study will help elucidate the carotenoid biosynthetic mechanism in *C*. *melo* and will provide valuable information for breeding chamoe cultivars with improved characteristics.

## 2. Materials and Methods

### 2.1. Plant Materials

Two chamoe cultivars, *Cucumis melo* L. var. *makuwa* ‘Ohbokggul’ and *C. melo* L. var. *makuwa* ‘Gotgam’, were grown in a greenhouse at an experimental farm, and obtained from Nongwoo Bio (Korea) during the fruiting season in October 2012. Ohbokggul and Gotgam chamoes are differentiated by shape, size, and color (Figure 1). Three fruits of each cultivar were collected, and their peels, pulps, and stalks were separated. The samples were frozen in liquid nitrogen and stored at −80 °C until analysis.

### 2.2. Isolation of cDNAs Encoding Carotenoid Biosynthetic Genes

GenBank accession numbers U38550, NM_125085, NM_001125948, NM_180954, and U58919 were used as queries to search for homologous sequences in our internal chamoe transcriptome database (unpublished data). Full-length cDNAs encoding ZDS, LCYE, CHXB, and ZEP, and partial-length cDNA encoding CHXE were isolated in *C. melo* and designated as CmZDS, CmLCYE, CmCHXB, CmCHXE, and CmZEP (GenBank accession numbers: KF668331, KF668332, KF668333, KF668334, and KF668335, respectively).

### 2.3. Quantitative Real-Time PCR Analysis

Quantitative RT-PCR was performed for the precise analysis of transcript levels. Primers targeted to CmPSY, CmPDS, CmLCYB, CmCCD1, CmNCED, and CmACT2 (Accession Nos. GU361622, KC507802, GU457407, XM_004170465, JF838293, and AB033599, respectively) and five genes isolated in this study were designed using the Primer Quest computer program (http://eu.idtdna.com/Scitools/Applications/Primerquest/), producing fragments of 80 to 90 bp (Table 1). Total RNA (5 μg) from each sample was combined with random hexamer primers in a SuperScript first-strand cDNA synthesis system according to the manufacturer’s instructions (Invitrogen Life Technologies, Carlsbad, CA, USA). After cDNA synthesis, quantitative real-time PCR was performed using SYBR^®^ Green SuperMix RT-PCR kit (IQ Sybr SYBR Green Super Mix, Bio-Rad, Hercules, CA, USA) on a MiniOption detection system (Bio-Rad, Hercules, CA, USA). Results were analyzed using Bio-Rad software (GeneXpression Macro Chromo4) and the comparative threshold cycle (Ct) method using CmACT2 as the reference according to the manufacturer’s instructions for data normalization.

### 2.4. Sequence Analysis

The deduced amino acid sequences of carotenoid biosynthetic genes from *C. melo* were analyzed for homology using the BLAST program and the NCBI GenBank database (http://www.ncbi.nlm.nih.gov/BLAST). Sequence alignments were carried out using BioEdit Sequence Alignment Editor, version 5.0.9 (Department of Microbiology, North Carolina State University, Raleigh, NC, USA). The predicted molecular mass of protein was calculated using an online website (http://www.sciencegateway.org/tools/proteinmw.htm).

### 2.5. Carotenoid Extraction and HPLC Analysis

Extraction and measurement of carotenoids by HPLC were performed as previously described by our group [24]. Briefly, carotenoids were released from the chamoe samples (0.02 g) by adding 3 mL of ethanol containing 0.1% ascorbic acid (*w*/*v*), vortex mixing for 20 s, and placing in a water bath at 85 °C for 5 min. The carotenoid extract was saponified with potassium hydroxide (120 μL, 80% *w/v*) in the 85 °C water bath for 10 min. After saponification, samples were placed immediately on ice, and cold deionized water (1.5 mL) was added. *β*-Apo-8′-carotenal (0.2 mL, 25 g/mL) was added as an internal standard. Carotenoids were extracted twice with hexane (1.5 mL) by centrifugation at 1200× *g* to separate the layers. Aliquots of the extracts were dried under a stream of nitrogen and redissolved in 50:50 (*v*/*v*) dichloromethane/methanol before analysis by HPLC. The carotenoids were separated on a C30 YMC column (250 × 4.6 mm, 3 μm; Waters Corporation, Milford, MA, USA) by Agilent 1100 HPLC (Massy, France) equipped with a photodiode array (PDA) detector. Chromatograms were generated at 450 nm. Solvent A consisted of methanol/water (92:8 *v*/*v*) with 10 mM ammonium acetate. Solvent B consisted of 100% methyl *tert*-butyl ether. Gradient elution was performed at 1 mL/min under the following conditions: 0 min, 90% A/10% B; 20 min, 83% A/17% B; 29 min, 75% A/25% B; 35 min, 30% A/70% B; 40 min, 30% A/70% B; 42 min, 25% A/75% B; 45 min, 90% A/10% B; and 55 min, 90% A/10% B. Carotenoid standards were purchased from CaroteNature (Lupsingen, Switzerland). For quantification, calibration curves were created by plotting four different concentrations of carotenoid standards according to the peak area ratios with *β*-apo-8′-carotenal. Quantification was performed using calibration curves ranging from 0.3 to 5 μg/mL. The linear equations were y = 0.1178x − 0.027 for zeaxanthin, y = 0.1194x − 0.0063 for lutein, y = 0.0822x − 0.0003 for β-carotene, y = 0.0822x − 0.0003 for 9-cis β-carotene, y = 0.0822x − 0.0003 for 13-cis β-carotene, y = 0.0822x − 0.0003 for α-carotene, and y = 0.0884x − 0.0251 for β-cryptoxanthin.

### 2.6. Statistical Analysis

The data on expression levels of carotenoid biosynthetic genes were analyzed using the computer software Statistical Analysis System (SAS version 9.2). Treatment means were compared by Duncan’s multiple range test.

## 3. Results

### 3.1. Sequence Analyses of Carotenoid Biosynthetic Genes from C. melo

CmZDS was composed of 1976 bp, with a 1731-bp open reading frame (ORF) encoding a protein of 576 amino acids (predicted molecular mass of 63.90 kDa; Figure S1). The closest homolog of CmZDS was ZDS from *Cucumis sativus* (98% identity and 99% similarity), followed by ZDS from *Cucurbita moschata* (93% identity and 96% similarity), ZDS from *Vitis vinifera* (89% identity and 95% similarity), and ZDS from *Citrus unshiu* (84% identity and 90% similarity). As shown in Figure S1, CmZDS contained a conserved dinucleotide-binding motif (GXGX_2_GX_3_AX_2_LX_3_GX_6_EX_5_GG) and a carotenoid-binding domain also found in other orthologous genes [25,26].

CmLCYE was 1958 bp long and had a 1602-bp ORF, encoding a protein of 533 amino acids with a predicted molecular mass of 58.81 kDa (Figure S2). CmLCYE shared 97% identity and 97% similarity with *Cucumis sativus* LCYE, 82% identity and 91% similarity with *Camellia sinensis* var. *assamica* LCYE, 83% identity and 90% similarity with *Glycine max* LCYE, and 79% identity and 88% similarity with *Vitis vinifera* LCYE. The deduced amino acid sequence of CmLCYE comprised a dinucleotide binding motif and two cyclase motifs, which are the common features of carotenoid cyclases [27,28].

CmCHXB consisted of 1292 bp with a 933-bp ORF and encoded a protein of 310 amino acids (predicted molecular mass of 34.78 kDa; Figure S3). CmCHXB exhibited 96% identity and 97% similarity with *Cucumis sativus* CHXB, 89% identity and 94% similarity with *Cucurbita moschata* CHXB, 77% identity and 86% similarity with *Vitis vinifera* CHXB, and 74% identity and 85% similarity with *Ipomoea nil* CHXB. Four conservatively spaced histidine motifs proposed to be involved in iron binding during hydroxylation reactions are marked in Figure S3 [29].

CmCHXE was composed of 933 bp encoding a partial 3′-end ORF of 148 amino acids. A BLAST search at the amino acid level showed that CmCHXE exhibited high homology to other CHXEs (Figure S4). Specifically, CmCHXE shared 97% identity and 97% similarity with *Cucumis sativus* CHXE, 84% identity and 92% similarity with *Vitis vinifera* CHXE, 88% identity and 94% similarity with *Fragaria vesca* subsp. *vesca* CHXE, and 83% identity and 93% similarity with *Daucus carota* subsp. *sativus* CHXE.

CmZEP was composed of 2514 bp with a 1998-bp ORF and encoded a protein of 665 amino acids with a predicted molecular mass of 73.20 kDa (Figure S5). CmZEP shared 98% identity and 98% similarity with *Cucumis sativus* ZEP, 95% identity and 96% similarity with *Citrullus lanatus* ZEP, 88% identity and 94% similarity with *Cucurbita moschata* ZEP, and 75% identity and 85% similarity with *Prunus armeniaca* ZEP. CmZEP displayed two short motifs typical of the lipocalin family of proteins and a phosphopeptide-binding domain (The forkhead-associated (FHA) domain), which are present in all known ZEP genes [30,31].

### 3.2. Expression Levels of Carotenoid Biosynthetic Genes in Ohbokggul and Gotgam Chamoes

**Figure 2:**
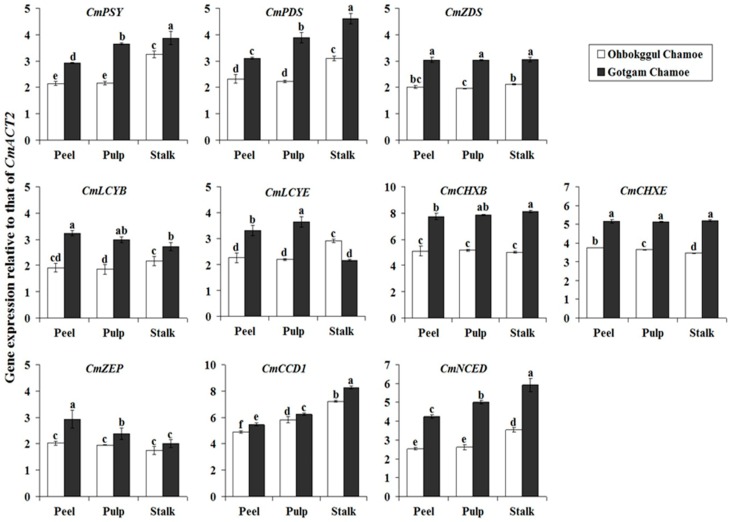
Expression levels of carotenoid biosynthetic genes in different parts of Ohbokggul and Gotgam chamoe fruit. Values are means; bars represent standard error from three independent measurements. The letters a, b, c, d, e, and f indicate significant differences at the 5% level by Duncan’s multiple range test.

Expression levels of carotenoid biosynthetic genes in the peel, pulp, and stalk of Ohbokggul were compared to those in Gotgam (Figure 2). In Ohbokggul chamoe, the highest expression levels of CmPSY were found in the stalk, with lower levels in the pulp and peel. This same pattern of expression was observed for *CmPDS*, *CmLCYB*, *CmLCYE*, *CmCCD1*, and *CmNCED*. Transcript levels of *CmCHXE* were highest in the peel and lowest in the stalk of Ohbokggul; *CmZDS*, *CmCHXB*, and *CmZEP* were expressed at similar levels in the peel, pulp, and stalk of Ohbokggul. In general, mRNA levels of carotenoid biosynthetic genes in all fruit parts were higher in Gotgam than in Ohbokggul. Transcription of most carotenoid biosynthetic genes (*CmPSY*, *CmPDS*, *CmCHXB*, *CmCCD1*, and *CmNCED*) was highest in the stalk and lowest in the peel of Gotgam chamoe. *CmLCYE* and *CmZEP* showed the highest expression levels in the pulp and peel, respectively. No differences in transcript levels of *CmZDS* and *CmCHXE* were found in the peel, pulp, or stalk of Gotgam chamoe.

### 3.3. Analysis of Carotenoid Content in Ohbokggul and Gotgam Chamoes

The same Ohbokggul and Gotgam peel, pulp, and stalk materials used for quantitative real-time PCR were used to analyze carotenoid composition and content by HPLC (Table 2). Surprisingly, carotenoids were very poorly synthesized in Ohbokggul fruit, with only trace amounts of total carotenoids measured in the peel (0.89 μg g^−1^), pulp (0.02 μg g^−1^), and stalk (0.51 μg g^−1^). In contrast, total carotenoid content was high in the peel of Gotgam chamoe (428.81 μg g^−1^). Lutein and β-carotene were the dominant compounds in Gotgam peel (278.05 μg g^−1^ and 112.02 μg g^−1^, respectively); lower concentrations of 9-*cis* β-carotene (10.27 μg g^−1^), 13-*cis* β-carotene (11.82 μg g^−1^), and β-cryptoxanthin (13.44 μg g^−1^) were also detected in the peel. Similar to the peel, lutein and β-carotene were the major carotenoids synthesized in the pulp, while only β-carotene showed an appreciable concentration in the stalk of Gotgam chamoe.

## 4. Discussion

In the present study, five carotenoid biosynthetic genes, *CmZDS*, *CmLCYE*, *CmCHXB*, *CmCHXE*, and *CmZEP*, were isolated from *C. melo*. Sequence analyses revealed that they shared high identity and common features with other orthologous genes. In addition, expression levels of entire genes involved in carotenoid biosynthetic pathways were investigated in different fruit parts of the Ohbokggul and Gotgam cultivars, the latter of which is a native Korean variety. *CmPSY*, which catalyzes the first committed and rate-limiting step in carotenoid biosynthesis [32,33], and most of the other carotenoid biosynthetic genes were expressed at their highest levels in the stalk. However, carotenoids were highly distributed in the peel, where tissue has direct exposure to light, suggesting the essential role of light in carotenoid accumulation in chamoe. On the other hand, PSY is often encoded by multiple genes which exhibit distinct expression and regulation in plants. There are two isoforms of PSY in tomato, where PSY1 is a chromoplast-specific isoform and PSY2 is a chloroplast-specific isoform [34]. It has been suggested that there is another isoform of *CmPSY* which directly regulates the carotenoid accumulation in the peel of chamoe. In addition, *CmCCD1*, which can cleave multiple carotenoid substrates at various positions, showed the highest expression level in the stalk [13]. Therefore, we hypothesize that the low content of carotenoids in the stalk of chamoe was because of the high activity of *CmCCD1* found in this part.

The expression levels of all carotenoid biosynthetic genes in Gotgam fruits were higher than those in Ohbokggul fruits, which probably led to the abundant carotenoid accumulation in Gotgam melons and the low carotenoid content in fruits of Ohbokggul. However, these higher expression levels of carotenoid biosynthetic genes cannot entirely account for the substantially higher total carotenoid content (up to 480-fold) in Gotgam peel compared to Ohbokggul peel. In addition, differences in carotenoid biosynthesis between the Ohbokggul and Gotgam cultivars provide a basic foundation for more detailed study of the molecular genetics of *C. melo*.

## 5. Conclusions

In conclusion, differences in the expression levels of carotenoid biosynthetic genes and carotenoid content between the cultivar Ohbokggul chamoe and the native Korean cultivar Gotgam chamoe were observed. These findings will contribute to a foundation for the elucidation of carotenoid biosynthesis in *C. melo*, an important commercial crop. In addition, further investigations regarding molecular genetics and enzyme activities may help to identify key genes for improving the carotenoid accumulation in *C. melo*.

## Figures and Tables

**Table 1:** Primers used in this study for quantitative real-time PCR analysis.

Gene Name	Primer Sequence (5′ to 3′)	
	Forward Primer	Reverse Primer
*CmPSY*	TGTGCAGAGTATGCCAAGAC	GTCCGCCTACACCATACATAAA
*CmPDS*	GGCTGGAGAAGTGGAGTTATTG	CCTCAGCTTAAAGCCAGAATACA
*CmZDS*	ACACTCCAGACGCAGATTTC	GCAATGATCCCTGTCCTTCA
*CmLCYB*	GTTTCTTCCCGAGCTGTTACT	GAGTTCCCTTTGCCATGATTTC
*CmLCYE*	TGGTCCAGATCTGCCATTTAC	CCGGCCATACATGCTCTATAC
*CmCHXB*	GCTGTCATGGCGGTTTATTAC	GGCACCAACAGAGAGAGAAA
*CmCHXE*	AATCGTTGCACTTGCCATATTC	GCTCCAGTAGTCATCCCAATG
*CmZEP*	GTAGAAGAATACGGGTTGCTGTA	CCGAGTCCAACTCCCAAATAA
*CmCCD1*	CATGATGAGACTCCTCCGATTAC	GATTTGGTCCCACCCTAACA
*CmNCED*	CAATCCTCTCTTCCAACCAACT	CTAGCGGAACCGTGATTGATAG
*CmACT2*	CTACGAACTTCCTGATGGACAAG	CCAATGAGAGATGGCTGGAATAG

**Table 2:** Carotenoid composition and content in different parts of Ohbokggul and Gotgam chamoe fruit (μg g^−1^ dry weight). The results are expressed as means ± standard error from three independent measurements. N.D., not detected.

Carotenoids	Ohbokggul Chamoe			Gotgam Chamoe		
	Peel	Pulp	Stalk	Peel	Pulp	Stalk
α-carotene	N.D.	N.D.	N.D.	2.54 ± 0.33	0.38 ± 0.01	N.D.
Lutein	0.45 ± 0.05	0.02 ± 0.00	0.07 ± 0.01	278.05 ± 23.51	14.16 ± 0.37	0.52 ± 0.11
β-carotene	0.27 ± 0.04	N.D.	0.33 ± 0.04	112.02 ± 10.69	6.45 ± 1.06	17.64 ± 3.94
9-*cis* β-carotene	0.02 ± 0.00	N.D.	0.02 ± 0.00	10.27 ± 0.69	0.50 ± 0.05	0.66 ± 0.15
13-*cis* β-carotene	0.07 ± 0.02	N.D.	0.04 ± 0.01	11.82 ± 1.56	0.96 ± 0.32	2.29 ± 0.53
β-cryptoxanthin	0.03 ± 0.01	N.D.	0.01 ± 0.00	13.44 ± 1.12	1.88 ± 0.19	2.23 ± 0.55
Zeaxanthin	0.05 ± 0.01	N.D.	0.05 ± 0.00	0.67 ± 0.07	0.11 ± 0.02	0.02 ± 0.00
Total	0.89 ± 0.14	0.02 ± 0.00	0.51 ± 0.07	428.81 ± 37.99	24.44 ± 2.01	23.35 ± 5.28

## Supplementary Materials

The following are available online at https://www.mdpi.com/2304-8158/8/2/77/s1, Figure S1: Multiple alignments of the amino acid sequences of CmZDS with other ZDSs. Figure S2: Multiple alignments of the amino acid sequences of CmLCYE with other LCYEs. Figure S3: Multiple alignments of the amino acid sequences of CmCHXB with other CHXBs. Figure S4: Multiple alignments of the amino acid sequences of CmCHXE with other CHXEs. Figure S5: Multiple alignments of the amino acid sequences of CmZEP with other ZEPs.
