# Supplementary material for: Carotenoid Biosynthesis in Oriental Melon (Cucumis melo L. var. makuwa)
Source: Foods. 2019 Feb 19;8(2):77. doi: 10.3390/foods8020077 (PMC6406825; doi:10.3390/foods8020077)
Supplement: Supplementary file 1 [file foods-08-00077-s001.pdf]

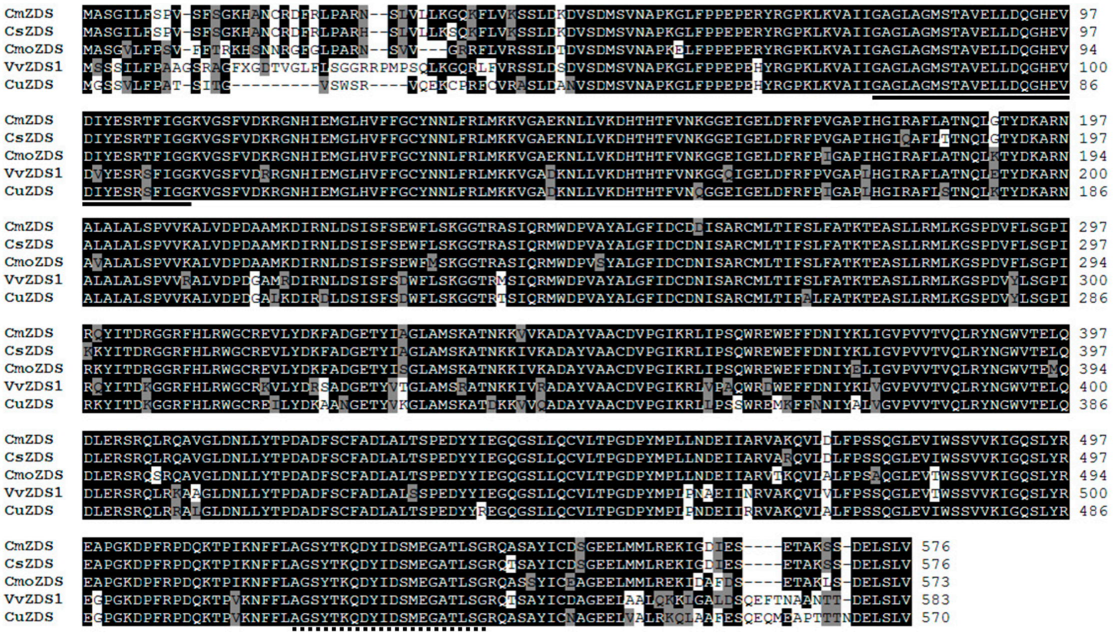

Figure S1. Multiple alignments of the amino acid sequences of CmZDS with other ZDSs. Identical residues are indicated by a black background, and similar residues are shaded with a gray background. The solid underline indicates the dinucleotide-binding domain, and the dotted underline indicates the carotenoid-binding domain. CsZDS, *Cucumis sativus* (XM_004142474); CmoZDS, *Cucurbita moschata* (JN253179); VvZDS1, *Vitis vinifera* (JQ319636); CuZDS1, *Citrus unshiu* (AB072343).

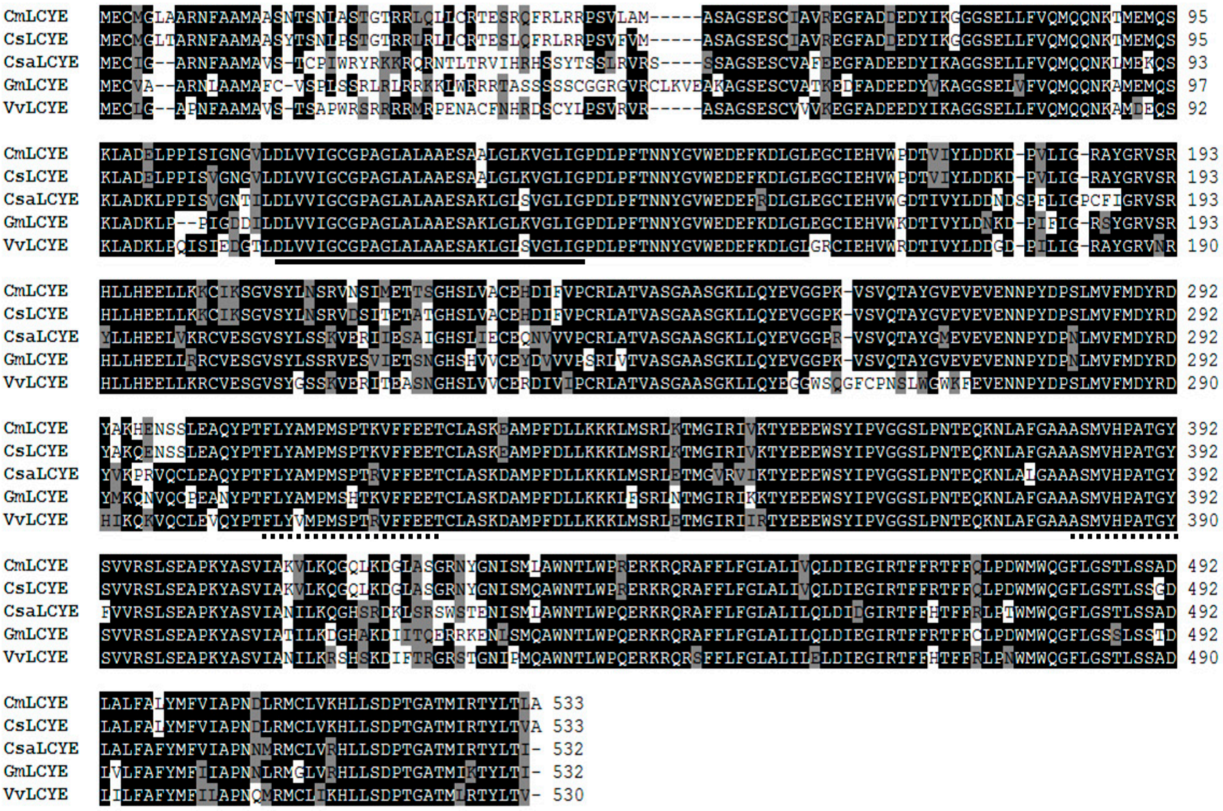

Figure S2. Multiple alignments of the amino acid sequences of CmLCYE with other LCYEs. Identical residues are indicated by a black background, and similar residues are shaded with a gray background. The solid underline indicates a dinucleotide binding motif, and the dotted underlines indicate two cyclase motifs. CsLCYE, *Cucumis sativus* (XM_004141124); CsaLCYE, *Camellia sinensis* var. *assamica* (HM536196); GmLCYE, *Glycine max* (XM_003533727); VvLCYE, *Vitis vinifera* (NM_001281164).

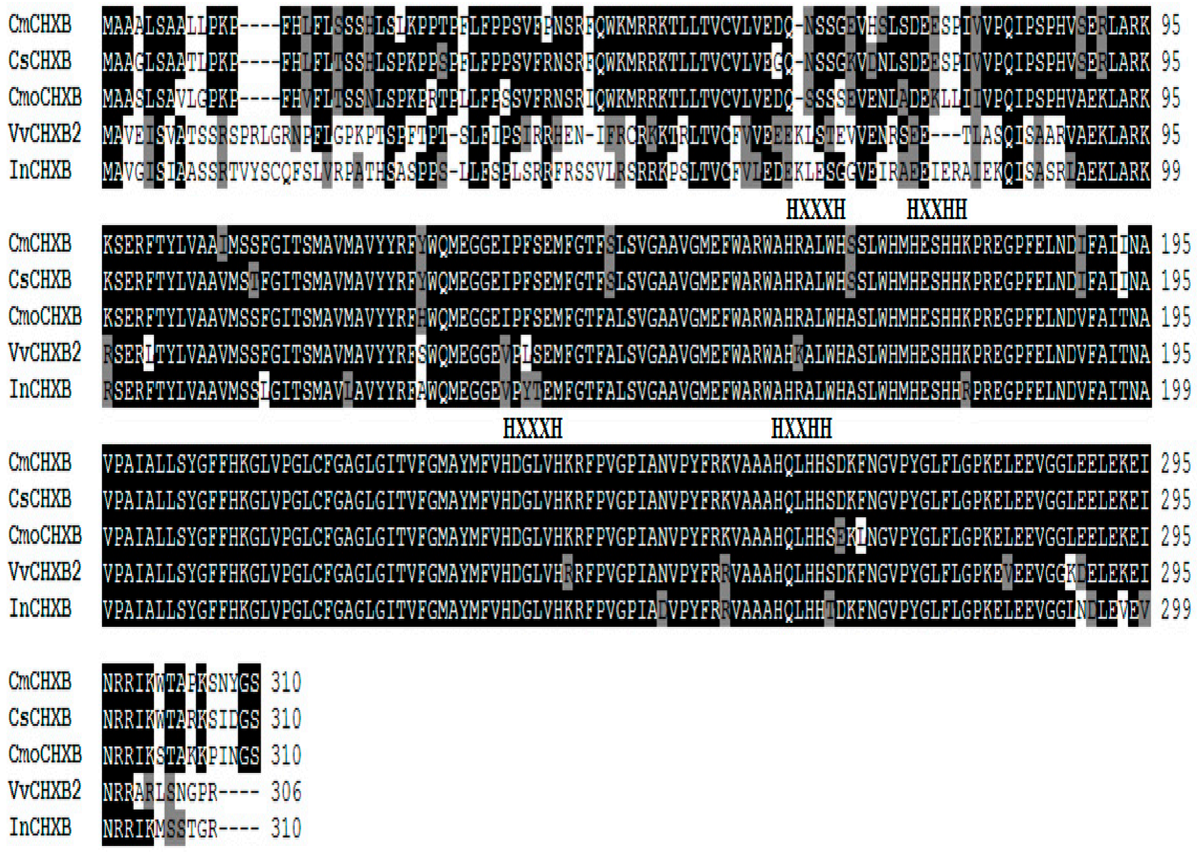

Figure S3. Multiple alignments of the amino acid sequences of CmCHXB with other CHXBs. Identical residues are indicated by a black background, and similar residues are shaded with a gray background. HXXXH and HXXHH, where X encodes for any amino acid, represent 4 conservatively spaced histidine motifs. CsCHXB, *Cucumis sativus* (XM_004140710); CmoCHXB, *Cucurbita moschata* (JN253180); VvCHXB2, *Vitis vinifera* (XM_002273545); InCHXB, *Ipomoea nil* (AB499058).

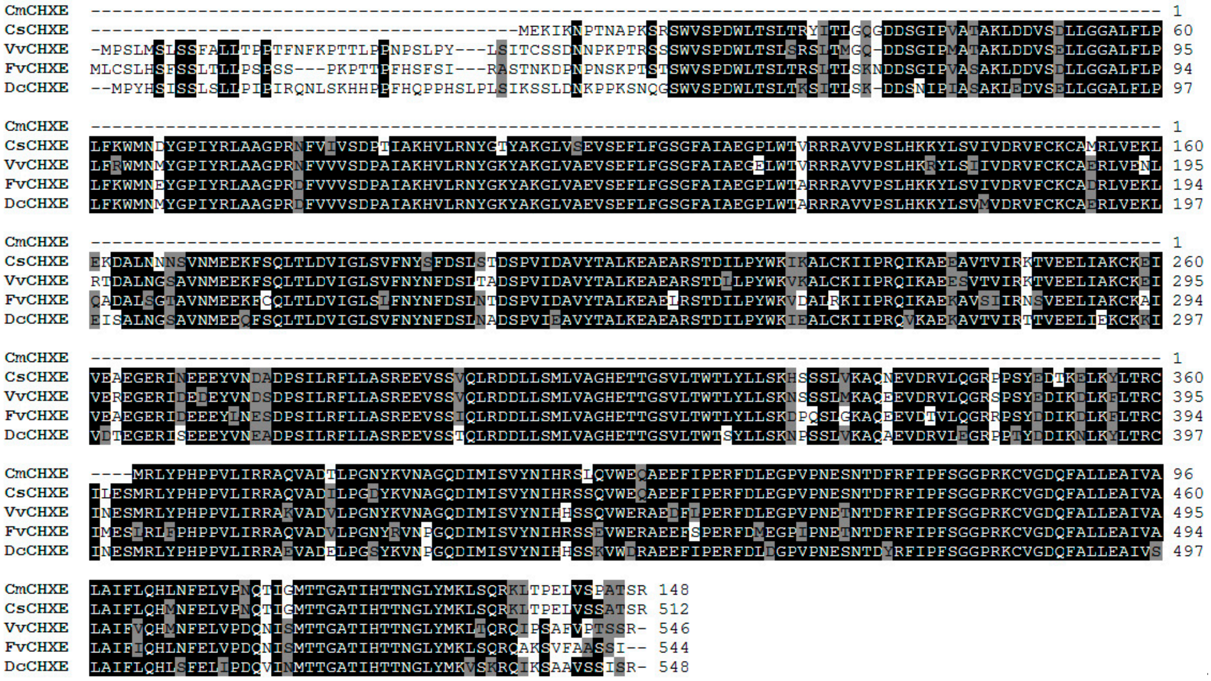

Figure S4. Multiple alignments of the amino acid sequences of CmCHXE with other CHXEs. Identical residues are indicated by a black background, and similar residues are shaded with a gray background. CsCHXE, *Cucumis sativus* (XM_004156280); VvCHXE, *Vitis vinifera* (XM_002264979); FvCHXE, *Fragaria vesca* (XM_004306122); DcCHXE, *Daucus carota* (DQ192196).

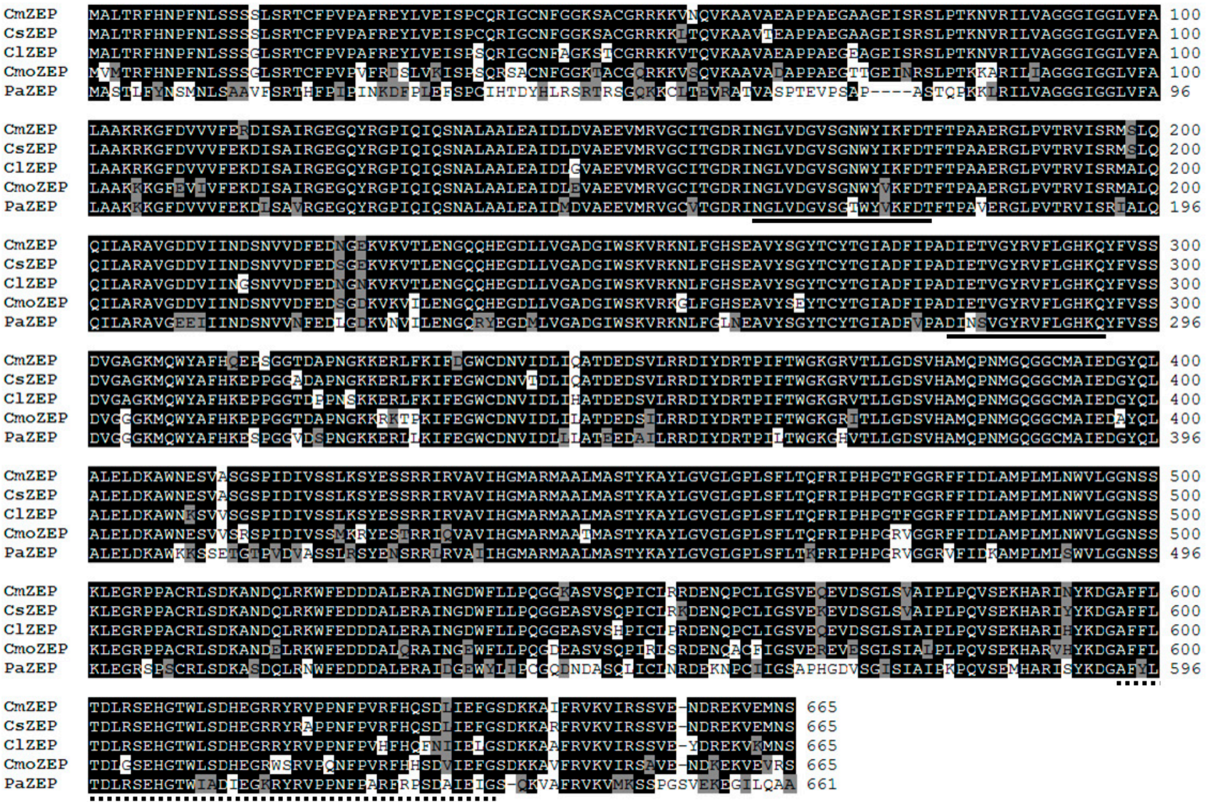

Figure S5. Multiple alignments of the amino acid sequences of CmZEP with other ZEPs. Identical residues are indicated by a black background, and similar residues are shaded with a gray background. The solid underlines indicate the short motifs typical of the lipocalin family of proteins, and the dotted underline indicates the FHA domain. CsZEP, *Cucumis sativus* (XM_004163228); ClZEP, *Citrullus lanatus* (HM107775); CmoZEP, *Cucurbita moschata* (JN581943); PaZEP, *Prunus armeniaca* (AF159948).
